# Marine-derived L-asparaginase: unlocking marine power in anti-tumor therapeutics

**DOI:** 10.3389/fimmu.2025.1710780

**Published:** 2025-12-12

**Authors:** Ritika Gopalakrishnan, Kamala Kannan, Ragul Gunasekaran, Priya Ramachandran, Dhanraj Ganapathy, Pitchiah Sivaperumal

**Affiliations:** 1 Centre for Marine and Aquatic Research (CMAR), Saveetha Institute of Medical and Technical Sciences, Saveetha University, Chennai, India; 2 Department of Prosthodontics, Saveetha Dental College and Hospitals, Saveetha Institute of Medical and Technical Sciences, Saveetha University, Chennai, Tamil Nadu, India

**Keywords:** marine-derived L-asparaginase, marine microorganisms, cancer therapy, acute lymphoblastic leukemia, combination therapy, autophagy, life below water

## Abstract

L-asparaginase, a critical enzyme for cancer therapy, has been primarily produced by microbes. Researches are being conducted to identify better stabilizing, low immunogenicity and highly active alternative sources has focused on marine microorganisms. Marine-derived L-asparaginase is a promising candidate due to its unique characteristics and broad application potential. This review discusses the molecular structure, production-related genes, and the search for marine microbial sources of L-asparaginase production. Roles of L-asparaginase in cancer metabolism including asparagine depletion, apoptosis induction, autophagy and immunity are also detailed. Clinical studies with L-asparaginase in the treatment of pediatric and adult acute lymphoblastic leukemia is described, with indications for solid tumors. Also, combination therapy using L-asparaginase such as chemotherapy, targeted therapy, and immunotherapy has been explored to enhance therapeutic effects. The discovery of marine-derived L-asparaginase variants with improved properties holds great potential for expanding the utility of this enzyme across multiple sectors, unlocking the marine power in cancer prevention.

## 1 Introduction

L-asparaginase (L-ASNase) is an enzyme in the amidohydrolase family (EC 3.5.1.1) that catalyzes the conversion of L-asparagine to L-aspartic acid and ammonia, a process crucial for the anti-cancer properties. It is a promising therapeutic enzyme used to treat acute lymphoblastic leukemia (ALL) and other hematological malignancies (1, 2). Although ASNases have a well-established role in food processing, particularly in reducing acrylamide levels, L-ASNase is primarily recognized in oncology for its ability to deprive cancer cells—particularly those auxotrophic for L-asparagine-a critical nutrient (3). It is used to treat a variety of cancer types, including acute myeloid leukemia (AML), lymphosarcoma, various non-Hodgkin’s lymphomas, and, most notably, acute lymphoblastic leukemia (ALL) (4). Since its clinical approval in 1978, L-asparaginase has become a standard treatment for acute lymphoblastic leukemia (ALL). This enzyme, primarily obtained from microbial sources, exhibits strong substrate specificity and catalytic efficiency, making it an effective agent against cancer cells that rely on external sources of L-asparagine for survival (5, 6). Commercially available L-asparaginases are derived from Bacteria such as *Escherichia coli* and *Erwinia chrysanthemi*. The enzyme’s biochemical roles and therapeutic possibilities are being widely studied in oncology to improve cancer therapy outcomes (7). Its mechanism of action includes depleting circulating asparagine, which selectively targets cancer cells that are auxotrophic for this amino acid and catalyzes the hydrolysis of asparagine to aspartic acid and ammonia, disrupting the metabolic pathways of cancer cells that rely on asparagine for growth (8, 9).

Asparagine metabolism plays a crucial role in distinguishing normal cells from cancerous cells, particularly in the context of amino acid utilization (10, 11). Cancer cells often exhibit auxotrophy for asparagine, relying on external sources for this amino acid, while normal cells can synthesize it effectively. This metabolic difference underpins therapeutic strategies targeting asparagine depletion, especially in hematological malignancies like ALL (12, 13). Asparagine is crucial for sustaining tumor cell growth, particularly in glutamine-depleted environments, where it compensates for the lack of glutamine (14). The enzyme asparagine synthetase (ASNS), essential for asparagine production, plays a significant role in this process, with its elevated activity linked to enhanced tumor invasiveness and metastasis (15). This is mediated through pathways such as Wnt signaling, which further promotes cancer progression (16). Asparagine availability directly impacts the translation of the MYC oncogene, a critical regulator of cell proliferation (17). Depletion of asparagine reduces MYC protein levels without affecting its mRNA, suggesting a post-transcriptional regulation mechanism (18). In glutamine-starved conditions, asparagine helps sustain c-MYC expression, which is essential for cellular viability and proliferation (19).

**Figure 1:**
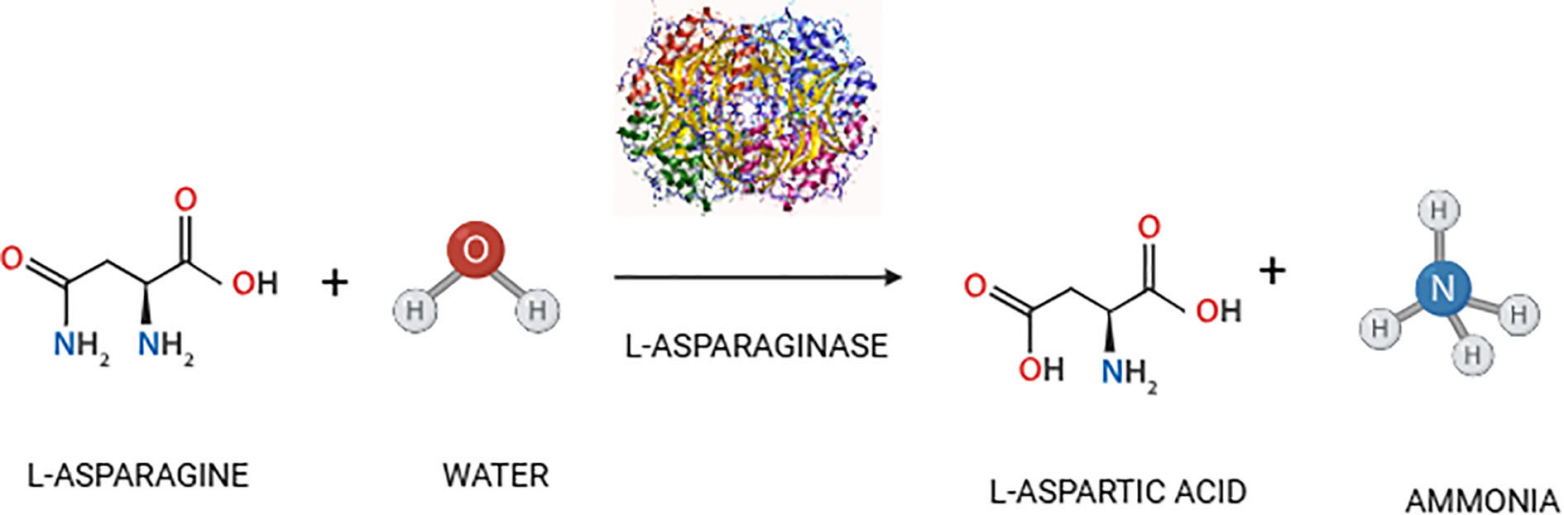
Catalytic reaction of L-asparaginase.

Marine bacteria, actinomycetes, fungi, and sponges thrive in extreme environments—from high salinity and pressure to low temperatures—conditions that naturally select for enzymes with enhanced biochemical robustness, including thermal stability, pH tolerance, and resistance to denaturation (20). Marine microorganisms thrive under unique physicochemical conditions—extreme salinity, pressure, and temperature—which endow their enzymes with remarkable structural resilience, stability, and catalytic efficiency. Such properties are highly desirable for biopharmaceutical enzymes, which must remain active in the complex and often hostile physiological environments of the human body (21). Moreover, marine-derived L-ASNases are believed to exhibit reduced immunogenicity, a critical parameter in clinical oncology, particularly for patients who develop hypersensitivity to currently approved L-ASNase formulations. This is supported by findings that some marine enzymes lack non-specific glutaminase activity, which is associated with toxicity in standard formulations (22). Marine-derived L-asparaginases, particularly those isolated from *Streptomyces*, *Bacillus*, and halophilic *Pseudomonas* species, are hypothesized to exhibit reduced immunogenic and toxic profiles compared to terrestrial counterparts. This proposition arises mainly from their low or absent glutaminase co-activity, which decreases off-target cytotoxicity and subsequent inflammatory immune activation. It is therefore more accurate to state that low glutaminase activity may reduce systemic toxicity, potentially leading to fewer secondary hypersensitivity reactions, rather than directly conferring lower antigenicity (23, 24). Marine enzymes are hypothesized to exhibit distinct active-site architectures or post-translational modifications, contributing to their lower allergenic potential. In addition to bacteria, marine sponges and associated symbiotic microbes are being investigated as unconventional but promising sources of L-ASNases and other bioactive molecules. These organisms have co-evolved in nutrient-scarce ecosystems, often relying on potent enzymatic pathways for survival and chemical defense, making them rich reservoirs of pharmacologically relevant enzymes (25). Despite these advantages, the transition from marine-source discovery to therapeutic application has been minimal. While terrestrial microorganisms such as *Escherichia coli* and *Erwinia chrysanthemi* have been widely studied and utilized in therapeutic settings, marine microorganisms remain significantly understudied despite offering substantial potential advantages. Few marine-derived L-ASNases have progressed beyond basic characterization, and no marine-origin variant has yet entered clinical trials for leukemia or solid tumor treatment. This underutilization may stem from challenges in isolating and culturing marine microbes, difficulties in expressing their enzymes recombinantly, and the lack of comprehensive structure-function studies to validate therapeutic viability. To bridge this gap, interdisciplinary approaches combining marine microbiology, genomics, structural biology, and protein engineering are needed. Techniques such as metagenomic screening, synthetic biology, and computational docking could accelerate the identification and optimization of marine L-ASNases with favorable pharmacokinetic and safety profiles (**Figure 1**).

## 2 Enzyme’s molecular structure

Structurally, L-asparaginase is typically a homotetramer, with bacterial-type L-asparaginases from *Escherichia coli* and *Erwinia chrysanthemi* being the most extensively studied forms (26). L-asparaginase is primarily known for its tetrameric form, though it can also occur as hexamers, dimers, and monomers, depending on its biological source (27). Molecular weight and secondary structure profiles vary among microbial sources. Most L-ASNase monomers fall between 35–55 kDa and exhibit a blend of α-helices and β-sheets, though some, like those from *Lactobacillus casei*, show predominantly helical structure (28). The future of ASNase research will likely focus on uncovering novel microbial enzymes—particularly from extremophiles—and using structure-guided mutagenesis to improve efficacy and safety in clinical and industrial contexts (29). Bacterial L-asparaginases generally exhibit comparable tertiary and quaternary structures, alongside shared biochemical properties (30). The enzyme functions as a homotetramer, with each monomer containing approximately 330 amino acid residues. The enzyme’s active site is situated between the N- and C-terminal domains of two adjacent monomers (31). This site is divided into a rigid segment, essential for ligand binding, and a flexible segment that regulates access to the binding pocket and contains the catalytic nucleophile, threonine-15 (Thr15), which is highly conserved across all L-asparaginases (32, 33).

An ASNase protomer is structured with a larger N-terminal domain (~195 amino acids) and a smaller C-terminal domain (~120 amino acids), connected by a 20–25 amino acid linker. N-terminal domain includes an eight-stranded β-sheet with α-helices Na1 and Na2 forming an active-site pocket and features a rare left-handed crossover between strands Nb4 and Nb5. Exposed helices Na1 and Na4 face the solvent, while Na2 and Na3 are near the domain interface (34). A β-hairpin (strands Nb7 and Nb8) orients toward the tetramer’s interior, and a unique disulfide bond (Cys77–Cys105) stabilizes the N-terminal sheet in EcAII. Another important feature is the flexible N-terminal loop, which can adopt open or closed conformations depending on substrate binding. This flexibility is key for forming the catalytically competent active site. Substrate binding induces closure of this loop, positioning residues like Thr12 and Tyr27 (in EcAII numbering) to facilitate nucleophilic attack and transition-state stabilization (35). The smaller C-terminal domain has a four-stranded parallel β-sheet with helices Ca3 and Ca4 on one side, while Ca1 and Ca2 are solvent-exposed at the domain apex (7, 36). The C-terminal domain of microbial L-ASNases contributes to substrate binding and structural integrity, typically consisting of a parallel β-sheet and flanking α-helices. While not directly catalytic, this domain is essential for dimer-dimer stabilization and influences enzyme solubility and folding. ASNases are generally classified as Type I (cytoplasmic) and Type II (extracellular), with the latter preferred in therapeutic settings due to higher substrate affinity (37). Enzymes derived from thermophilic and marine bacteria, such as *Thermococcus kodakarensis* and *Melioribacter roseus*, display enhanced thermal and pH stability and reduced glutaminase activity—qualities desirable for pharmaceutical and industrial applications (38).

L-asparaginase derived from *E. carotovora* is unique in that it is composed of two tetramers (ABCD and EFGH), totaling eight monomers (A through H). Each tetramer comprises four identical subunits, effectively forming a “dimer of dimers.” In each monomer, 327 amino acids organize into a structure with 14 α-strands, eight β-helices, and the typical N- and C-terminal domains (39). The active form of all bacterial L-asparaginases is described as homotetramers of about 130 to 140 kDa, usually referred to as a dimer of dimers, with four identical non-cooperative active sites formed at the intimate dimer interface (40). The enzyme’s active site is formed at the interface between adjacent monomers (specifically A and C, or B and D) through a precise arrangement of amino acids. One monomer contributes residues such as Thr15, Tyr29, Ser62, Glu63, Thr95, Asp96, Ala120, and Lys168, with Thr15 and Thr95 playing key catalytic roles, while the neighboring monomer includes only Ser254 in this region (41). Interestingly, Pseudomonas L-asparaginases exhibit structural similarities to other bacterial amidohydrolases, except for a distinctive 20-residue loop, which is part of their active site (39). This unique arrangement supports the enzyme’s catalytic function and is fundamental to its ability to degrade asparagine, an amino acid critical for cancer cells. The active site is located at the interface between subunits, where specific residues ensure substrate specificity and contribute to the enzyme’s catalytic efficiency. In some cases, structural mutations, such as the substitution of Tyr278 with methionine in *Bacillus licheniformis* L-asparaginase, have revealed critical residues affecting substrate binding and catalytic action (42). In *Escherichia coli* type II L-asparaginase, structural studies reveal a tightly packed active site formed by residues from adjacent subunits. These structural features limit unwanted side activities, like glutaminase activity, enhancing its therapeutic efficacy for targeting leukemic cells. Moreover, mutations such as N24S stabilize the enzyme’s active site configuration, which is critical for maintaining activity in clinical contexts (43). Recent macromolecular investigations have revealed distinctive structural motifs in newly characterized L-asparaginase variants that directly contribute to enhanced substrate affinity and minimized glutaminase co-activity (44). High-resolution crystallographic and computational modeling analyses demonstrated that subtle amino acid substitutions within the catalytic loop and β-sheet framework can drastically modify the geometry of the active site, improving substrate binding efficiency while maintaining structural rigidity. These conformational refinements reduce nonspecific glutamine hydrolysis—one of the principal limitations of currently marketed asparaginase formulations—thereby enhancing therapeutic specificity and lowering cytotoxic side effects. Moreover, hydrogen-bonding adjustments and hydrophobic core stabilization were found to increase enzyme thermostability and solvent tolerance, traits highly desirable for clinical and biotechnological application.

The structural arrangement and the conserved biochemical properties of L-asparaginase make it a valuable therapeutic enzyme. Its quaternary structure, stability, and conserved active site enable its specific binding and catalytic activity, which have been exploited in oncology, particularly in treating acute lymphoblastic leukemia.

## 3 Genes involved in L-asparaginase production

The ansA and ansB genes in *Escherichia coli* and *Erwinia chrysanthemi* encode L-asparaginase enzymes, essential for converting L-asparagine to aspartate and ammonia, thus supporting nitrogen utilization under limited nitrogen conditions (45). Encoded by the ansA gene, Type I L-asparaginase is constitutively expressed under normal growth conditions. In *Escherichia coli*, the *ansA* gene encodes a Type I L-asparaginase that is constitutively expressed and exhibits low substrate affinity, functioning primarily under nutrient-rich conditions as part of general nitrogen metabolism. In contrast, the *ansB* gene encodes the Type II isoenzyme, which is inducible under nitrogen-limited conditions and has higher affinity for asparagine, making it the therapeutically relevant form used in clinical applications. Similarly, in marine actinomycetes like *Streptomyces griseus* NIOT-VKMA29, the ansA gene has been identified and heterologously expressed in *E. coli*, resulting in a 3-fold increase in L-ASNase activity, highlighting its centrality in enzyme biosynthesis (46). It has low substrate affinity and is primarily involved in general metabolism rather than nitrogen scavenging or therapeutic applications (47). The ansB gene encodes Type II L-asparaginase, which has a much higher affinity for asparagine and is significantly induced under nitrogen-limiting conditions. This enzyme is widely used in the pharmaceutical industry due to its effectiveness in asparagine depletion, which starves cancer cells (48). ansB is inducible and encodes the high-affinity isoenzyme that has therapeutic significance due to its higher activity at physiological L-asparagine concentrations (27).

Expression of the ans genes is tightly regulated by environmental cues and controlled by several transcriptional regulators. In *E. coli*, crp (cyclic AMP receptor protein) and fnr (fumarate and nitrate reduction regulator) are among the primary global regulators that indirectly influence ansB transcription by modulating cellular responses to oxygen and carbon source availability (49, 50). Additionally, AnsR, a repressor protein, binds to the ansB promoter in the absence of asparagine and prevents unnecessary enzyme synthesis. Upon substrate availability, AnsR repression is lifted, allowing gene expression to proceed (51).

The ansA gene is vital for nitrogen metabolism in E. coli, especially in environments where external nitrogen is scarce. This gene enables cells to use L-asparagine as a nitrogen source (52). On the other hand, ansB encodes an asparaginase with unique regulatory mechanisms and enzymatic properties, further contributing to nitrogen balance within the cell under various environmental conditions. Studies have shown that these genes help cells adapt to nitrogen-poor environments by promoting asparagine breakdown when other nitrogen sources are unavailable (53, 54). Both ansA and ansB are upregulated in nitrogen-limiting conditions, regulated by nitrogen-sensing mechanisms that adjust enzyme levels to meet cellular demands. This regulation is influenced by other factors like oxygen and carbon sources, with aerobic conditions in *E. coli* favoring the efficient utilization of L-asparagine in nitrogen-poor environments. The adaptive response of these genes under nitrogen scarcity has implications for optimizing L-asparaginase production, which is valuable in medical and industrial applications (55).

The genetic pathways regulating L-asparaginase (L-ASNase) production in *E. coli* and *Erwinia chrysanthemi* involve complex responses to nitrogen levels, largely controlled by the NtrB/NtrC system. This two-component system is vital under nitrogen-limited conditions, where NtrB activates NtrC, which then promotes transcription of ansA and ansB, the genes encoding L-ASNase. Recent research shows that nitrogen limitation not only activates NtrC but also coordinates broader metabolic responses, including the synthesis of amino acids and nitrogen uptake, helping bacteria adapt to varying nitrogen levels (56, 57). This system also impacts resistance to oxidative stress, suggesting a complex interaction between nitrogen regulation and cellular stress responses. In addition to nitrogen regulation, global regulators like the cAMP receptor protein (CRP) influence ansA and ansB expression in response to carbon source availability, integrating multiple environmental cues into L-ASNase production pathways (58). CRP functions by binding to cAMP, a secondary messenger whose levels increase when glucose is scarce. Under glucose-limiting conditions, CRP-cAMP complexes activate the transcription of genes involved in alternative nutrient metabolism, including *ansB *(59). This regulation allows *E. coli* to prioritize glucose metabolism when it is available while inducing *ansB* expression during nitrogen starvation or when asparagine becomes a crucial nitrogen source. The interaction between CRP and the *ansB* promoter ensures that L-asparaginase is produced only under conditions where its function is advantageous for cellular survival (60). Besides transcriptional regulation, proper post-translational processing and protein folding are essential for functional L-asparaginase activity. Chaperone proteins such as DnaK, GroEL, and Trigger Factor (TF) assist in the correct folding of nascent L-ASNase chains. In recombinant expression systems, co-expression of these chaperones with ans genes has been shown to increase the soluble yield of the active enzyme, especially in hosts like *E. coli* and *Bacillus subtilis (*61). Additionally, signal peptides, particularly in ansB, direct the enzyme to the periplasm or extracellular space, facilitating secretion. Modifications such as N-terminal methionine cleavage and disulfide bond formation also influence enzyme stability and activity, particularly in high-temperature or pH-stressed environments (62).

While bacterial genes often occur in operons, recent studies have shown that eukaryotic fungi also harbor clustered metabolic genes, including those for ASNase. Unlike operons, these clusters are typically not polycistronic, but they are co-regulated and often found near transcription factors or resistance genes. In *Fusarium*, *Penicillium*, and *Trichosporon* species, ASNase biosynthetic genes are co-localized with genes involved in stress response and nitrogen metabolism, forming functionally interlinked clusters. This “quasi-operon” organization facilitates coordinated expression in response to environmental triggers like nitrogen depletion or oxidative stress (63, 64). In actinomycetes like *Streptomyces griseus*, genomic sequencing has revealed operons containing ansA-like genes, flanked by promoters responsive to nitrogen limitation and sigma factors that modulate environmental stress responses. Novel ans-like genes have been discovered in marine and extremophilic microorganisms, revealing genetic diversity with promising therapeutic properties. Shirazian et al., have reported that in isolates from halophilic environments such as Urmia Salt Lake, several L-asparaginase genes encoding glutaminase-free variants have been reported. The clinical significance of low glutaminase activity lies primarily in reduced off-target cytotoxicity, not necessarily in elimination of antigenicity. Mechanistically, glutaminase co-activity causes depletion of glutamine in non- tumor tissues, which can lead to hepatocellular injury, pancreatitis and increased oxidative stress; these tissue insults release damage-associated molecular patterns (DAMPs) and pro-inflammatory cytokines that promote antigen presentation and adaptive immune priming (65). Supporting this trend, Luhana and Patel (2013) isolated three halophilic bacterial strains—designated HJK1, HJK2, and HJK3—from Bet Dwarka marine waters, which exhibited strong extracellular L-asparaginase activity without detectable glutaminase or urease contamination. Although gene-level data were not reported, the enzymatic characteristics indicated the presence of high-affinity, low-toxicity L-asparaginases, typical of ansB-type isoenzymes. This observation reinforces the hypothesis that halophilic bacteria, due to adaptive genomic configurations under saline stress, may express modified asparaginase isoforms with naturally reduced immunogenic co-activities (66). Such native selectivity makes halophilic isolates promising for cloning and further genetic characterization of their L-asparaginase loci. Thus, an L-asparaginase with negligible glutaminase activity is hypothesized to produce a milder inflammatory milieu and reduce the likelihood of toxicity-driven immune sensitization. Similarly, thermophilic variants from *Melioribacter roseus* and marine actinomycetes exhibit codon-optimized gene sequences adapted for expression at high temperatures and alkaline pH, making them ideal for industrial or oral formulations. These discoveries are guiding synthetic biology approaches to engineer ans genes with site-directed mutagenesis or modular cloning for enhanced pharmacokinetics, reduced immunogenicity, and broader substrate selectivity (67). The results highlighted that marine extremophiles can generate L-asparaginases comparable or superior to conventional *E. coli* and Erwinia chrysanthemi formulations, particularly in terms of stability and catalytic efficiency.

Studies have shown that nitrogen levels can alter the expression of hundreds of genes, including those directly related to L-ASNase, reflecting a broad adaptive response to nutrient stress. Optimizing these conditions could enhance L-ASNase production in microbial systems, as recent findings emphasize how transcription factors coordinating nitrogen metabolism significantly impact yield (68).

## 4 Exploring marine microorganisms for L-asparaginase production

While commercial L-ASNase production primarily relies on *Escherichia coli* and *Erwinia chrysanthemi*, marine microorganisms have recently gained attention as promising alternative sources of this enzyme (69). The primary difference between *E. coli* and *E. chrysanthemi* L-ASNase lies in immunogenicity and activity. The principal clinical difference between *Escherichia coli*–derived and *Erwinia (Erwinia chrysanthemi)*–derived L-asparaginases lies in their immunogenic profiles and pharmacokinetics (70). Native *E. coli* asparaginase and pegylated formulations are associated with higher and more variable rates of clinical hypersensitivity (reported ranges typically 10–30% in many series, with older reports up to ~75% in specific cohorts), whereas *Erwinia* formulations have historically been used as alternatives in patients who develop anti-E. coli antibodies and often show lower cross-reactivity and different hypersensitivity frequencies in clinical practice (71). While both sources are effective in depleting asparagine, E. coli L-ASNase often shows higher immunogenicity, leading to hypersensitivity reactions. Consequently, E. chrysanthemi-derived L-ASNase is commonly used in patients with allergies to E. coli-based formulations (72, 73). Additionally, *E. chrysanthemi* L-ASNase has a shorter half-life, which can necessitate more frequent dosing but offers an alternative for patients with immune responses to E. coli preparations (74). Recent recombinant advancements, such as JZP-458 derived from *Pseudomonas fluorescens*, also aim to replicate *E. chrysanthemi’*s immunogenic benefits while enhancing stability (75).

Similarly, marine bacteria isolated from unique environments show diverse biochemical properties, offering the potential to isolate L-ASNase variants with enhanced stability and reduced side effects (76). An in-silico prediction on endophytic bacteria have also gained attention, as they may produce L-ASNase with favorable immunological indices that could reduce allergic reactions in patients (77). Key factors enhancing the therapeutic efficacy of L-ASNase include enzyme stability, reduced immunogenicity, and selectivity. Stability is essential for maintaining enzyme activity in physiological conditions, and researchers are employing genetic engineering to enhance L-ASNase thermal and pH stability (78). Furthermore, reducing immunogenicity remains a priority, as immune reactions can compromise treatment efficacy. PEGylation, as used with *E. coli* asparaginase, significantly reduces immunogenic responses and prolongs enzyme circulation time, though it may not completely prevent immune reactions (79, 80). Selectivity is another crucial factor; enzymes with minimal activity on glutamine are preferred to avoid off-target effects and toxicity. Structural analysis has revealed that *E. coli* and *E. chrysanthemi* L-ASNases differ in their active site configurations, impacting their glutaminase activity and selectivity (43).

Marine microorganisms such as Pseudomonas, Bacillus, Streptomyces, and Aspergillus exhibit unique metabolic characteristics, potentially yielding novel L-ASNase enzymes with improved stability, reduced immunogenicity, and higher efficacy in diverse environmental conditions (81). The unique properties of L-ASNase from these sources, including enhanced stability, activity in various conditions, and reduced immunogenicity, suggest they could complement or replace conventional enzyme sources in therapeutic and industrial settings. Pseudomonas species, particularly *P. aeruginosa*, are widely recognized for their metabolic versatility and ability to produce L-ASNase. Research into *Pseudomonas aeruginosa* CSPS4, isolated from marine environments, revealed that this strain produces a thermo-acidophilic form of L-ASNase, exhibits optimal catalytic activity around pH 6 and 60 °C. Importantly, the enzyme retains substantial activity over a broader range of pH (5–9) and temperatures (30–70 °C), indicating notable thermal and pH stability. This broad stability profile makes it particularly suitable for industrial processes such as acrylamide reduction in the food industry (82). In Pseudomonas aeruginosa, although ansA-like genes are also present, emerging research has focused on broader regulatory proteins such as AmrZ, a transcription factor that impacts multiple metabolic pathways, including cellulose biosynthesis and possibly asparaginase expression under biofilm-forming and oxidative stress conditions (83). Additional studies on Pseudomonas strains suggest their potential for therapeutic applications, given the enzyme’s unique structural properties and low immunogenicity (84). Marine-derived Bacillus species have also shown promise for L-ASNase production. In marine Bacillus species, such as *Bacillus niacini* and *Bacillus altitudinis*, L-asparaginase genes are typically part of a broader nitrogen utilization operon, often regulated by nitrogen starvation signals (85). Moreover, regulatory motifs within promoter regions respond to nutrient levels and environmental pH, contributing to the enzyme’s functional resilience across industrial conditions. For instance, *Bacillus niacini* isolated from marine saltern sediments demonstrated significant extracellular L-ASNase activity, with stability under a range of temperatures and pH levels (86). *Bacillus altitudinis*, isolated from marine crabs, produced an extracellular L-ASNase with minimal glutaminase and urease activity, enhancing its therapeutic safety profile. This strain’s enzyme exhibited high stability, a favorable Michaelis constant (Km), and nonallergenic properties, making it a strong candidate for clinical applications was analyzed through in-silico analysis (87).

The genus Streptomyces, known for producing bioactive compounds, has been extensively studied as a marine source of L-ASNase. For example, *Streptomyces griseus* NIOT-VKMA29, isolated from marine sediment, was found to produce L-ASNase at high yields, with optimization techniques enhancing its activity. Molecular analysis revealed base substitutions in the ansA coding sequence compared to terrestrial Streptomyces, resulting in amino acid changes that enhance enzyme stability and activity (46). Furthermore, Streptomyces from marine sponges were found to carry diverse secondary metabolite biosynthesis gene clusters (BGCs), including L-ASNase-related genes. Horizontal gene transfer events appear to have enriched these clusters, allowing Streptomyces to adapt to nutrient-limited and competitive marine environments (88). Many L-asparaginase genes in marine organisms may remain cryptic under standard culture conditions. For example, in *Streptomyces ansochromogenes*, a global regulator called AdpA represses certain biosynthetic genes, including cryptic clusters for secondary metabolites. Disruption of adpA was shown to activate previously silent gene clusters, a strategy that could be extended to uncover cryptic L-ASNase genes in other marine actinomycetes (89). In a related study, *Aspergillus niger* was identified as a promising producer of extracellular L-asparaginase with strong anticancer potential (90). The purified enzyme exhibited optimum activity at pH 8.0 and 40 °C, showing potent cytotoxicity against lymphoblastic cells while maintaining stability under storage and physiological conditions. Importantly, its extracellular secretion simplifies downstream purification and reduces production cost, highlighting filamentous fungi as economically viable sources. When compared to marine and extremophilic counterparts, such terrestrial fungal models offer a valuable biochemical benchmark, demonstrating the importance of merging conventional fungal biotechnology with marine enzyme engineering for enhanced pharmacological outcomes. Moreover, novel transcriptional regulators like LmbU in *Streptomyces lincolnensis* have been identified as direct activators of specific biosynthetic genes, and manipulating such regulators via synthetic biology can enhance enzyme yields in engineered strains (91).

In addition to bacterial sources, marine fungi such as Aspergillus and other filamentous fungi show potential as L-ASNase producers. In marine fungi, such as *Aspergillus oryzae* and *A. flavus*, L-asparaginase production is controlled by homeobox-domain transcription factors, notably Hbx1 and HbxA, which regulate the expression of hundreds to thousands of downstream genes, including those in secondary metabolite biosynthesis (92). Transcriptomic analyses in *A. flavus* and *A. nidulans* have shown that deletion of hbx1/hbxA leads to suppression of both developmental and metabolic genes, some of which are co-regulated with putative L-ASNase homologs (93, 94). Recently, Luhana et al. (2025) achieved successful purification and detailed characterization of Lasparaginase derived from *Aspergillus flavus* HK03, marking a significant step toward developing fungal sources for therapeutic enzyme production (92). The enzyme exhibited a molecular weight of approximately 78 kDa, with optimum catalytic activity at pH 7.0 and 37 °C, and kinetic constants of Km ≈ 0.061 mM and Vmax ≈ 67.98 U/mL. Beyond its robust biochemical profile, the enzyme displayed pronounced anti-proliferative activity against human leukemic cells, achieving an IC_₅₀_ value near 2.5 U/mL (95). These findings highlight *A. flavus* as a promising biocatalyst producer with dual therapeutic and industrial relevance. The stability and specificity demonstrated by this fungal asparaginase suggest that *Aspergillus* species could serve as potential candidates for sustainable, low-immunogenic formulations. *Aspergillus oryzae*, commonly used in fermentation, has been observed to produce L-ASNase with properties suitable for industrial applications (96). Moreover, optimization studies using *Beauveria bassiana*, a marine fungus, indicate that L-ASNase production can be enhanced through response surface methodology, achieving optimal activity in controlled media (97). Expanding the horizon of fungal asparaginases, Luhana and Bariya (2023) conducted a comparative analysis of purified anti-leukemic L-asparaginase derived from *Trichoderma* species. Their work revealed Km values of approximately 0.0528 mM and 0.0565 mM for two strains, with corresponding Vmax values of 55.24 U/mL and 71.13 U/mL, indicating strong substrate affinity and catalytic efficiency. The enzyme displayed high stability across a wide temperature and pH range and demonstrated potent cytotoxicity toward leukemic cells (98). Such findings highlight the adaptability of *Trichoderma* and related fungal genera as dependable enzyme sources, capable of producing high-activity, low-toxicity asparaginases suitable for therapeutic and industrial applications. The inclusion of marine-adapted fungal strains within this research trajectory may further enhance enzyme stability and expand the therapeutic landscape for L-asparaginase. Maximizing enzyme yield and stability is crucial for L-ASNase’s therapeutic and industrial applications. Recent studies exploring fungal biodiversity in unique regions like Yemeni soil have revealed high-producing ASNase strains from genera such as *Penicillium*, *Fusarium*, and *Aspergillus*. Importantly, some strains produce L-ASNase without associated glutaminase or urease activity, enhancing their therapeutic potential. These fungal isolates likely express gene clusters uniquely adapted for nitrogen metabolism and detoxification, which are genetically regulated by nutrient-sensing transcription factors and external pH (99, 100). These discoveries pave the way for future metagenomic mining, cluster engineering, and CRISPR-based regulation to harness novel L-ASNase genes from underexplored marine ecosystems.

Further exploration of uncharted marine environments, such as deep-sea sediments and unique ecosystems, may yield novel strains with high enzyme activity and unique properties. Recent explorations of halophilic bacterial communities from the Bet Dwarka marine ecosystem have highlighted their potential as promising reservoirs of bioactive enzymes, particularly Lasparaginase. Isolates from this region demonstrated notable extracellular enzyme activity with favorable catalytic efficiency and stability under physiological conditions. These halophilic variants exhibit optimal function at neutral to slightly alkaline pH and moderate temperatures, features that enhance their suitability for therapeutic application (66). Combining traditional isolation techniques with next-generation sequencing could accelerate the discovery and characterization of marine-derived L-ASNase enzymes for clinical and industrial use (101). Continued research into marine-derived L-ASNase, coupled with optimization and genetic modification techniques, holds great potential for expanding the utility of this enzyme across multiple sectors.

## 5 Involvement in metabolic pathways of cancer

**Figure 2:**
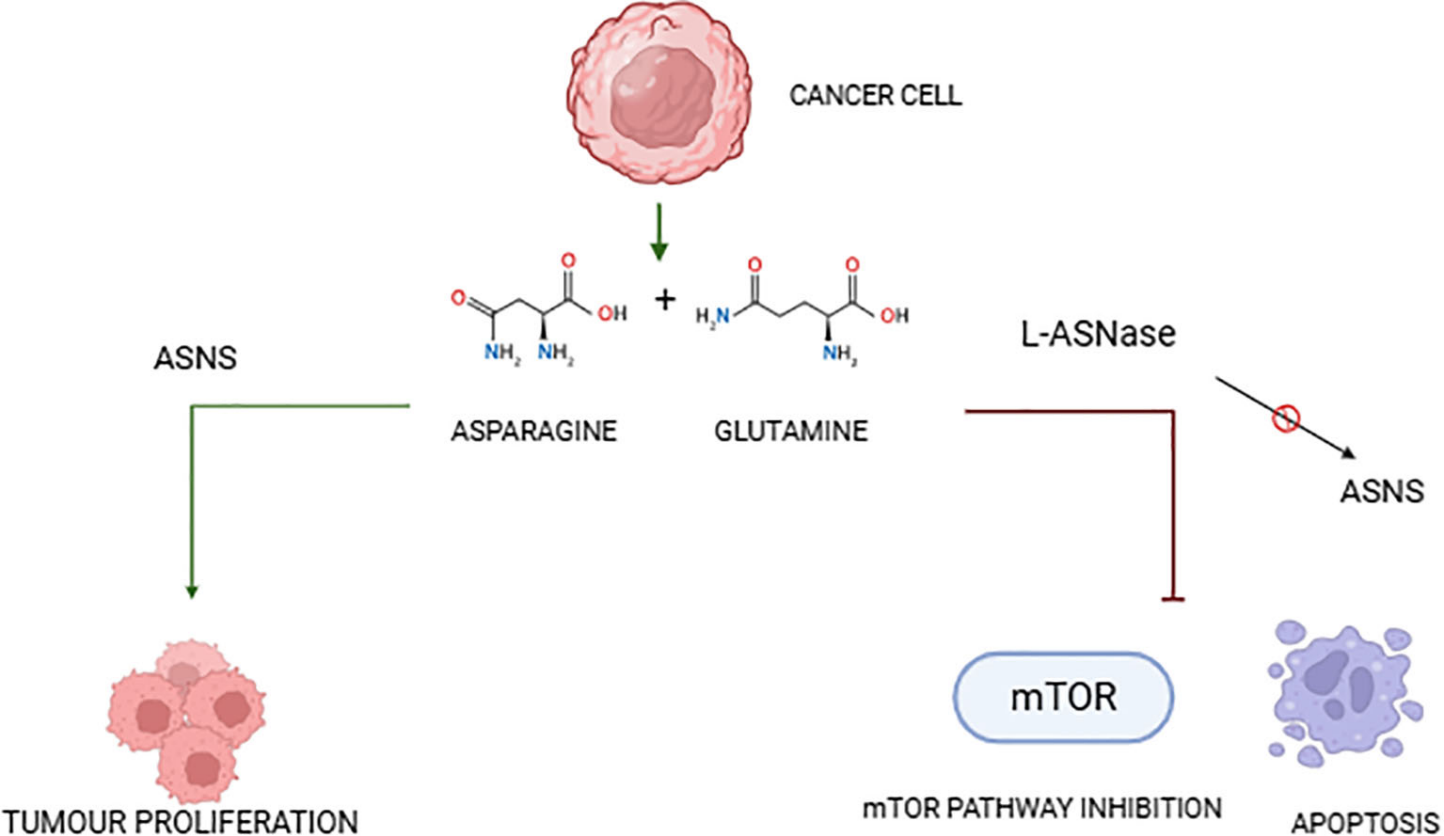
Schematic representation of L-asparaginase action and its impact on tumor cells.

L-asparaginase (L-ASNase) is a critical enzyme in cancer treatment, particularly in managing acute lymphoblastic leukemia (ALL). Its mechanism revolves around depleting the amino acids asparagine and, to some extent, glutamine, which creates metabolic stress that leads to cancer cell apoptosis through several cellular pathways, particularly influencing the mTOR pathway, autophagy, and metabolic processes (102). L-Asparaginase (L-ASNase) exerts its cytotoxic effect by depleting extracellular asparagine, exploiting cancer cells’ reliance on external sources due to their low expression of asparagine synthetase (ASNS). This metabolic disruption triggers amino acid starvation responses, leading to multiple cellular stress pathways that culminate in apoptosis (12, 15). NF-κB is a crucial regulator of inflammatory and anti-apoptotic pathways, and its downregulation sensitizes cancer cells to apoptosis by inhibiting the expression of survival genes such as cIAP1, cIAP2, and survivin (103). Concurrently, caspase-3/7 activation is a hallmark of L-ASNase-induced apoptosis, as seen in dose-dependent studies where increased annexin-V positive cells correlate with decreased procaspase-3 levels (104). Dalisay et al. described that marine-derived L-ASNases, particularly those isolated from *Streptomyces* sp., induced mitochondrial-mediated apoptosis in colorectal carcinoma cell lines through activation of caspase-3/9 and cytochrome c release (25). The depletion of asparagine disrupts protein folding and translation, initiating unfolded protein response (UPR) and ER stress-mediated apoptotic signaling. Further, asparagine deprivation induces reactive oxygen species (ROS) accumulation, leading to oxidative damage and mitochondrial membrane depolarization. Zuo & Kwok demonstrated that marine enzymes not only perturb amino acid metabolism but also interfere with glycolytic flux and tricarboxylic acid (TCA) cycle intermediates, leading to metabolic exhaustion and autophagic collapse (22) (**Figure 2**).

### 5.1 Asparagine depletion and apoptosis induction

L-asparaginase depletes asparagine, amino acid essential for protein synthesis and cellular metabolism. Cancer cells, particularly ALL cells, rely heavily on extracellular asparagine due to limited asparagine synthetase (ASNS) expression (105). The depletion of asparagine leads to a decline in substrate availability for protein and nucleotide synthesis, resulting in significant metabolic stress and apoptosis. The depletion of asparagine induces apoptosis, as cancer cells are unable to maintain protein synthesis without sufficient amino acids, leading to metabolic stress and cell death (2, 106). Moreover, glutamine depletion impacts the glutamate and glutathione pools, which are crucial for cellular redox balance and energy production, pushing cancer cells toward apoptosis (107). Mitochondrial dysfunction plays a pivotal role in the apoptotic cascade induced by L-ASNase. The depletion of asparagine and glutamine leads to reactive oxygen species (ROS) accumulation, which exacerbates oxidative stress within cancer cells (108). This process is further intensified when autophagy, a cytoprotective mechanism, is inhibited. Studies show that mitochondrial ROS accumulation enhances Bax translocation to the mitochondria, triggering the release of cytochrome C and promoting caspase activation (109).

L-Asparaginase (L-ASNase) significantly alters immune cell metabolism, indirectly affecting the tumor microenvironment. Immune cells, particularly T cells and macrophages, require distinct metabolic pathways to function effectively (110). Under normal conditions, effector T cells (Teffs) rely heavily on glycolysis for rapid energy production, while regulatory T cells (Tregs) and memory T cells favor oxidative phosphorylation (OXPHOS) and fatty acid oxidation (FAO) to sustain long-term immune responses. The depletion of asparagine and glutamine by L-ASNase disrupts these metabolic pathways, leading to impaired immune responses (14, 111). The metabolic shift caused by L-ASNase leads to T cell exhaustion, as activated T cells depend on glycolysis for energy and function. The lack of asparagine affects mTORC1 signaling, which is crucial for T cell activation, differentiation, and cytokine production. This downregulation of mTORC1 leads to reduced interferon-gamma (IFN-γ) production and impaired immune surveillance against tumors (112). Additionally, glutamine depletion disrupts the glutamate-glutathione balance, increasing oxidative stress in immune cells. This metabolic imbalance weakens antitumor immunity, allowing cancer cells to evade immune attack. However, some studies suggest that targeting Tregs with L-ASNase could reduce immunosuppression in the tumor microenvironment, enhancing the efficacy of immunotherapies such as checkpoint inhibitors (107, 113).

### 5.2 Autophagy

In tumor cells, L-asparaginase induces a specific autophagic response alongside apoptosis. Research demonstrates that the enzyme activates the apoptosis-related protein caspase-3 and triggers autophagy pathways evidenced by autophagosome formation, thus enhancing its cytotoxic effect on CML cells (114). L-asparaginase effectively inhibits the mechanistic target of rapamycin complex 1 (mTORC1), which regulates cell growth and metabolism. This pathway is sensitive to amino acid levels, and depletion of asparagine by L-ASNase disrupts mTORC1 signaling by affecting the activation of Rag GTPases necessary for mTORC1 activation. The inhibition of mTORC1 reduces protein synthesis and cell growth, contributing to cancer cell apoptosis (115). By depleting asparagine and glutamine, L-ASNase reduces the phosphorylation of mTORC1 targets, such as p70S6K and 4E-BP1, which are essential for protein translation. This downregulation of mTOR signaling contributes to decreased protein synthesis and cell growth, promoting apoptosis in ALL cells (116). Additionally, mTOR inhibition by L-asparaginase also impacts autophagy. Autophagy, a process typically induced by nutrient deprivation, helps cells recycle components during stress. By inhibiting mTOR, L-asparaginase enhances autophagy in cancer cells, which may serve as a cytoprotective mechanism in some cancers (117). Studies on glioblastoma, for instance, show that blocking autophagy while administering L-asparaginase enhances cancer cell death, as autophagy inhibition prevents the cancer cells from mitigating the metabolic stress induced by asparagine depletion (118).

The interaction between L-asparaginase and autophagy is complex. While L-asparaginase induces apoptosis, it simultaneously activates cytoprotective autophagy pathways, which cancer cells may use to resist therapy. The suppression of autophagy through pharmacological inhibitors like chloroquine has been found to increase the efficacy of L-asparaginase by preventing cells from recycling damaged components, thereby increasing cell stress and promoting apoptosis, indicating that autophagy acts as a protective mechanism in certain cancer types, such as glioblastoma and CML (119, 120). Several preclinical studies have examined L-asparaginase (L-ASNase) activity in glioblastoma models. Early work showed that L-ASNase induces metabolic reprogramming and cell-death signaling in glioma cell lines and patient-derived glioma cultures (*in vitro*), and that combining L-ASNase with autophagy inhibition potentiates tumor growth suppression in xenograft (*in vivo*) models. For example, Chen et al. demonstrated L-ASNase–induced autophagy and apoptosis in U87MG and U251MG glioblastoma cells (*in vitro*) and reported enhanced tumor growth inhibition when autophagy was pharmacologically blocked in U87 xenografts (*in vivo*). These results indicate that autophagy is an inducible response to asparagine deprivation in glioblastoma models and that modulation of autophagy can alter therapeutic outcomes (118).

In leukemia and other cancers, L-asparaginase treatment also influences immune responses by affecting metabolic regulation. For instance, depletion of asparagine modulates immune function by indirectly affecting immune cell metabolism, thereby influencing the tumor microenvironment. This modulation has the potential to enhance the effectiveness of immunotherapies by making the cancer cells more vulnerable to immune-mediated attack (14, 112, 121). The mitogen-activated protein kinase (MAPK) pathway is another crucial regulator of cell survival that is significantly impacted by L-ASNase. The MAPK signaling cascade, particularly the RAS-RAF-MEK-ERK axis, is frequently activated in cancers to promote tumor growth, survival, and resistance to therapy (122). L-ASNase treatment disrupts this pathway by decreasing ERK phosphorylation, leading to reduced transcription of pro-survival genes, including Bcl-2 and c-Myc. This disruption enhances caspase-3 activation, promoting apoptosis. Additionally, MAPK pathway inhibition sensitizes cancer cells to oxidative stress, as L-ASNase-induced mitochondrial ROS accumulation further amplifies apoptosis (123). Importantly, resistant cancer cells often compensate for MAPK inhibition by activating parallel survival pathways such as PI3K/AKT/mTOR, suggesting that dual inhibition of mTOR and MAPK signaling in combination with L-ASNase could be an effective strategy to prevent resistance. Combination therapies with MEK inhibitors (trametinib) or mTOR inhibitors (rapamycin) have shown synergistic effects in preclinical models, highlighting potential translational applications (124).

In rapidly proliferating cancer cells, glycolysis is the preferred metabolic pathway, even in the presence of oxygen (Warburg effect). This reliance on glycolysis provides cancer cells with ATP and metabolic intermediates required for biosynthesis (125). However, when glycolysis is inhibited due to asparagine depletion, cancer cells activate AMP-activated protein kinase (AMPK), which enhances FAO to compensate for energy loss (126). L-asparaginase profoundly affects cellular metabolism by promoting FAO and reducing glycolysis, thereby inducing metabolic stress. This shift from glycolysis to FAO reduces the cell’s energy supply, contributing further to apoptosis in cancer cells (127, 128). In leukemic cells, L-ASNase increases the reliance on FAO while downregulating glycolytic pathways, leading to a metabolic imbalance. Inhibition of FAO in combination with L-asparaginase treatment has been shown to enhance cancer cell sensitivity to apoptosis, as the cells cannot compensate for the loss of glycolytic energy production (129). Under nutrient stress, leukemic cells often turn to alternative amino acid pathways, such as glutamate, to sustain their growth. Blocking glutamate uptake can amplify the cytotoxic effects of L-ASNase, as cells are left with fewer resources to adapt to amino acid deprivation. This inhibition disrupts glutathione synthesis, which is critical for managing oxidative stress, thus increasing cancer cell susceptibility to L-ASNase (130, 131).

## 6 Clinical trials involving L-asparaginase

### 6.1 Pediatric and adult ALL trials

L-Asparaginase (L-ASNase) has been extensively studied as a treatment for hematological malignancies, primarily acute lymphoblastic leukemia (ALL). Clinical research highlights its distinct efficacy in pediatric ALL and explores adaptations in adult populations to manage higher rates of toxicity and immunogenicity (132). L-ASNase has achieved high efficacy in pediatric ALL, notably improving event-free survival rates when combined with agents like vincristine and corticosteroids. Studies report up to 90% event-free survival in pediatric cases with L-ASNase (133). However, adult trials reveal increased toxicity. Pegylated L-asparaginase (pegaspargase) formulations are being trialed to improve tolerability, but response rates are still lower in adults (134). Recent studies explore genetically engineered L-ASNase variants to mitigate adverse effects while enhancing efficacy. An example is the development of thermoresponsive polypeptide-fused L-ASNase, which provides longer stability, reduced immunogenicity, and enhanced efficacy in models of leukemia and lymphoma (135). Besides ALL, L-ASNase shows potential in treating other hematologic cancers, such as lymphomas. Ongoing research is examining the role of L-ASNase in combination with oncolytic viruses or CAR-T cell therapies for broader therapeutic application (136). The use of L-Asparaginase (L-ASNase) in treating solid tumors is emerging, with research focusing on specific cancers like ovarian, breast, and melanoma. L-ASNase is well-known for its success in hematologic cancers, and recent studies explore its efficacy in solid tumors (137). In a study on ovarian cancer, L-ASNase, particularly in its pegylated form, was shown to hinder tumor cell adhesion, presenting an anti-metastatic effect and opening avenues for targeting ASNS-deficient ovarian tumors (138). Research on breast cancer models indicates that L-ASNase efficacy depends on dosing schedules and circadian rhythms, with optimal timing improving anti-tumor effects. Studies using murine breast cancer models show that administration timing significantly impacts plasma asparagine levels, supporting L-ASNase as an effective treatment if applied strategically (139). L-ASNase effectiveness in melanoma cells was noted to be limited by the upregulation of ASNS, which some tumor cells utilize to counteract asparagine deprivation. Blocking ASNS in melanoma models amplified L-ASNase efficacy, emphasizing ASNS expression as a predictive biomarker for sensitivity (140). CRISPR screenings have highlighted resistance mechanisms in melanoma, suggesting combination therapies could be a way to optimize outcomes (106). Tumors with low ASNS expression are particularly vulnerable to L-ASNase, as they depend on extracellular asparagine. In contrast, high ASNS levels confer resistance, suggesting ASNS expression as a key biomarker (141). For instance, studies have shown that ASNS inhibition combined with L-ASNase enhances cell cycle arrest and apoptosis in certain resistant tumors like melanoma, epidermoid carcinoma, triple-negative breast cancer, KRAS-mutant colorectal and lung cancers, ovarian cancer, supporting its role as a therapeutic target (142).

**Table 1:** Clinical trials and studies on L-asparaginase (L-ASNase).

S.No	Trial studies	Patient group	L-ASNase formulation	Key findings	Source
1	Acute Lymphoblastic Leukemia (ALL)	Pediatric	Pegaspargase	High event-free survival (>90%) in pediatric ALL; well-tolerated	(143)
2	Acute Lymphoblastic Leukemia (ALL)	Adult	Pegaspargase, native ASNase	Pediatric-inspired regimens show improved survival in Adolescent and young adults; toxicity management remains crucial	(134, 144)
3	ALL (T/B Cell)	Pediatric & Adult	Genetically engineered L-ASNase (low glutaminase activity)	New variants maintain efficacy and show reduced toxicity	(145)
4	Acute Lymphoblastic Leukemia (ALL)	Pediatric	L-ASNase pulse and intensified therapy	Ongoing Japanese nationwide trial evaluating risk-adapted therapy with L-ASNase	(146)
5	Acute Lymphoblastic Leukemia (ALL)	Adult	Pegaspargase	Guidelines developed to optimize dosing and manage hypersensitivity, hepatotoxicity, etc.	(147)
6	Acute Lymphoblastic Leukemia (ALL)	Pediatric & Adult	General use	Toxicities include hypersensitivity, thrombosis, pancreatitis; early management improves outcomes	(148)
7	Ovarian Cancer	Solid Tumor Models	Pegylated L-ASNase	Inhibits tumor adhesion and metastasis in ASNS-deficient cells	(138)
8	Breast cancer	Murine models	Native L-ASNase	Timing of dosing affects efficacy via circadian rhythm modulation	(139)
9	Melanoma	Preclinical	L-ASNase + ASNS inhibition	High ASNS expression induces resistance; co-targeting ASNS enhances therapy	(106, 142)

This table summarizes key clinical and preclinical studies involving L-Asparaginase (L-ASNase) across hematological malignancies and solid tumors. It includes information on patient populations, formulations used (e.g., native, pegylated, or genetically engineered L-ASNase), and major findings from the studies. Trials cover pediatric and adult applications in acute lymphoblastic leukemia (ALL), as well as exploratory research in ovarian, breast, and melanoma cancer models.

### 6.2 Combination therapies with L-asparaginase

#### 6.2.1 Combination with chemotherapy

Combining L-Asparaginase (L-ASNase) with other therapies is increasingly explored to improve cancer treatment outcomes. These combination strategies utilize chemotherapy, targeted therapies, and immunotherapy to enhance L-ASNase’s effectiveness, especially against resistant cancers (149). L-ASNase shows synergistic effects when paired with agents like cytarabine or anthracyclines. Synergistic interactions between L-ASNase and cytotoxic agents like cytarabine, methotrexate, and gemcitabine help overcome resistance and improve response rates (112). For example, gemcitabine, a nucleoside analog, has demonstrated synergy with immune checkpoint inhibitors (ICIs) in mesothelioma and preclinical models by depleting immunosuppressive cells and promoting antigen release (150). This synergy occurs because each agent targets different cancer pathways, helping to reduce resistance. Similar synergistic potential exists when pairing L-ASNase with such agents, as asparagine depletion creates metabolic stress that sensitizes tumor cells to DNA-damaging agents (151). In non-small cell lung cancer (NSCLC), chemotherapy combined with PD-1/PD-L1 inhibitors significantly improved progression-free survival, paving the way for integrating L-ASNase in future combinatorial protocols (152). Studies on lung cancer indicate that chemotherapy combined with immunotherapy outperforms monotherapies, showing the potential for L-ASNase to play a role in multi-agent chemotherapy protocols (153).

#### 6.2.2 Integration of immunotherapy and nutrient starvation

Tumor progression and anti-tumor immunity are tightly coupled to cellular metabolic networks, and mounting evidence positions asparagine as a critical metabolic nexus influencing both cancer cell survival and T-cell function (154). L-ASNase was originally developed to exploit the asparagine auxotrophy of acute lymphoblastic leukemia, recent mechanistic and translational studies demonstrate that transient asparagine depletion remodels the tumor microenvironment (TME) in ways that can potentiate CD8^+^ T-cell responses and increase sensitivity to PD-1/PD-L1 blockade (155). Mechanistically, limiting extracellular asparagine shifts T-cell metabolic programming. Preclinical data indicate that moderate asparagine restriction enhances mitochondrial fitness of activated CD8^+^ T cells, increases oxidative phosphorylation capacity, and promotes effector cytokine production such as IFN-γ and granzyme B—phenotypes associated with superior tumor control and resistance to exhaustion (156). Tumor-intrinsic asparagine metabolism also mediates immune evasion. High tumor expression of asparagine synthetase (ASNS) maintains local asparagine pools and is associated with suppression of type I interferon signaling and impaired antigen-presentation pathways. Conversely, ASNS knockdown or systemic asparagine depletion reduces tumor growth in a CD8^+^ T-cell-dependent manner in several models, supporting a model in which tumor asparagine metabolism functions as a modifiable immune-evasion axis (157). Thus, targeting asparagine availability has the dual potential to directly impair tumor cell fitness and to relieve tumor-intrinsic immunosuppression. L-ASNase is increasingly combined with immune checkpoint inhibitors to promote a more hostile environment for tumor cells. A recent study with a long-acting form of L-ASNase, ASNase-ELP, combined with a PD-1 inhibitor, showed superior efficacy in models of metastatic solid tumors (158). This approach, termed “starvation-immunotherapy,” depletes asparagine, sensitizing cancer cells to immune-mediated destruction (159). A notable strategy involves using a long-acting variant of L-ASNase (ASNase-ELP) in combination with PD-1 blockade, achieving durable tumor regression in metastatic models. These strategies are supported by studies in hepatocellular carcinoma (HCC), where dual immunotherapy (anti-PD-L1/CTLA-4) with molecular agents improved antitumor response in patients with otherwise limited options (160, 161). Researchers have observed increased infiltration of polyfunctional CD8^+^ T cells (those that can produce multiple cytokines), a decrease in immune-suppressive myeloid cells, and the reactivation of interferon signaling inside tumor cells (162). Early compassionate-use reports from patients also show promising outcomes when L-ASNase is given alongside PD-1 inhibitors, suggesting potential synergy even in cases where checkpoint therapy alone had failed (163). Combination therapies using L-ASNase and other anticancer agents offer promising strategies for managing resistant cancers. By integrating chemotherapy, targeted therapies, and immunotherapies, L-ASNase efficacy can be amplified while also reducing the emergence of resistance (14). Although immunogenicity is often discussed alongside enzyme-related toxicities, these two properties arise from distinct mechanisms. The immunogenicity of L-asparaginase is primarily influenced by sequence-derived epitopes that trigger antibody production, whereas its systemic toxicity is largely attributed to off-target glutaminase activity and subsequent metabolic stress. Elevated glutaminase co-activity can indirectly enhance immune activation by causing hepatic injury, oxidative stress, and cytokine release, thereby amplifying inflammatory responses to the therapeutic enzyme (164). Marine-derived L-asparaginases typically exhibit low or negligible glutaminase activity, resulting in reduced cytotoxicity and a milder inflammatory environment. This biochemical property may lower secondary immune sensitization, but it does not inherently eliminate antigenicity, which still depends on the enzyme’s amino acid composition and structural epitopes (165).

#### 6.2.3 Enhancing efficacy via autophagy inhibition

One of the resistance mechanisms triggered by L-ASNase is autophagy, which allows cancer cells to recycle intracellular nutrients under stress. Inhibiting autophagy alongside L-ASNase enhances its cytotoxicity (120, 166). For instance, chloroquine, an FDA-approved antimalarial agent, is a widely used autophagy inhibitor. It disrupts lysosomal acidification, halting autophagic flux. In breast cancer models, chloroquine-loaded nanoparticles combined with PD-L1 antibodies and chemotherapeutics induced apoptosis and reversed immune-cold tumor microenvironments (167). Autophagy inhibition increases reactive oxygen species (ROS) accumulation, which in combination with L-ASNase-mediated nutrient stress, overwhelms cancer cell defenses. Beyond autophagy, L-ASNase induces mitochondrial dysfunction, triggering apoptosis via calcium dysregulation and ROS-mediated mitochondrial permeability transition pore (mPTP) opening (168). Combining L-ASNase with mitochondrial-targeting agents such as bcl-2 inhibitors (e.g., venetoclax) or agents affecting mitochondrial metabolism intensifies apoptosis. Mitochondrial priming enhances the release of pro-apoptotic factors like cytochrome c, activating caspase cascades. These effects are synergistically amplified when paired with ICIs, which recruit T cells that secrete IFN-γ, further disrupting mitochondrial function in cancer cells. Studies in ovarian and colorectal cancer suggest combining mitochondrial stress inducers with immune therapies leads to better control over micrometastatic disease (169, 170). The interplay between ROS accumulation and autophagy inhibition suggests that combination therapies targeting autophagy pathways, such as the use of chloroquine or hydroxychloroquine, may enhance L-ASNase-induced cytotoxicity (171). Moreover, as mitochondrial stress builds up, mitochondrial permeability transition pore (mPTP) formation further amplifies apoptosis by causing calcium dysregulation and loss of mitochondrial membrane potential (172). Personalizing L-ASNase-based combination therapy hinges on tumor genomics, metabolic phenotype, and immune environment. Translational studies increasingly integrate L-ASNase into multi-modal regimens, including chemotherapy, radiation, checkpoint blockade, and metabolic inhibitors. For example, in non-small cell lung cancer (NSCLC) and ovarian cancer, checkpoint inhibitors have limited standalone efficacy; however, combinations with L-ASNase and other agents targeting DNA repair (e.g., PARP inhibitors) are under investigation for synergistic cytotoxicity (173, 174).

## 7 Future perspectives

Future research should concentrate on exploring marine microorganisms to find novel sources of L-ASNase with improved stability, less side effects, and more efficacy. Understanding the environmental factors driving enzyme production in these organisms could reveal new strategies for enzyme optimization. With advances in genome analysis and genetic engineering, researchers can improve the enzyme’s properties and alter treatments based on cancer types and patient needs. The use of omics technologies, such as metabolomics and transcriptomics, could provide deeper insights into the mechanisms of L-ASNase action and resistance pathways in cancer cells. There is also a great potential to expand L-ASNase use beyond blood cancers to solid tumors by combining it with other treatments, like immunotherapy to overcome resistance and improve outcomes.

Reducing side effects, especially allergic reactions, is a ultimate priority. This can be achieved by developing new enzyme formulations, such as pegylated or encapsulated versions, to make treatments safer. In addition to its medical applications, marine-derived L-ASNase could be used in industries like food processing to reduce harmful chemicals. Advanced technologies like AI can speed up the discovery and development of better L-ASNase enzymes, making them more effective and widely useful in medicine and industry. Advances in AI and machine learning provide new opportunities for enzyme modeling and optimization, thereby speeding up the development of L-ASNase. These technologies can predict the stability and efficacy of an enzyme potential to trigger side effects. By combining quick testing methods with advanced computer analysis, researchers can more easily find the best enzyme candidates for medical or industrial use.

## 8 Conclusions

L-asparaginase (L-ASNase) is at the center and complex enzyme in the field of cancer treatment and especially in the specific therapeutic control of acute lymphoblastic leukemia (ALL). Its mechanism of action is by depletion of amino acids asparagine and glutamine, cellular deep metabolic stress that results in cancer cell apoptosis by a variety of complex cellular processes (e.g., mTOR pathway, autophagy). The effectiveness of L-ASNase in pediatric ALL has been well established, including studies reporting spectacular event free survival rate up to 90% in the pediatric forms. However, adult trial data implicate a rise in toxicities and/or adverse effects and, accordingly, pegylated forms of L-asparaginase formulation, as well as genetically engineered L-asparaginase have been investigated to minimize the toxicity, and to improve the therapeutic efficacy. In addition to its proven use in hematologic malignancies, L-ASNase has been further investigated as an anti-tumor therapy for solid tumors in other cancer series including ovarian, breast and melanoma cancers. Further, the study of L-ASNase/combinatorial therapies with other anticancer drugs is a promising therapeutic means for resistant cancers, and the synergistic effect of L-ASNase together with chemotherapy, targeted therapy and immunotherapy has been proven. With multi-functional characteristic of L-ASNase in cancer treatment and the promising function of L-ASNase in the different cancers and the synergistic effects of L-ASNase combined therapy, it highlights the critical role of L-ASNase in the current cancer treatment, and thus offers the window for improved therapeutic strategies and patient management in cancer.
